# Cervical lymph node carcinoma metastasis from unknown primary site: a retrospective analysis of 154 patients

**DOI:** 10.1002/cam4.1458

**Published:** 2018-04-02

**Authors:** Yan Wang, Sha‐Sha He, Yong Bao, Xiu‐Yu Cai, Hai‐Yang Chen, Xing‐Li Yang, Dan‐Ming Chen, Li‐Xia Lu, Yong Chen

**Affiliations:** ^1^ Department of Radiation Oncology The First Affiliated Hospital of Sun Yat‐Sen University Guangzhou China; ^2^ Department of Radiation Oncology Sun Yat‐Sen University Cancer Center Guangzhou China; ^3^ Department of VIP Region Sun Yat‐Sen University Cancer Center Guangzhou China; ^4^ Department of Radiation Oncology The Sixth Affiliated Hospital of Sun Yat‐Sen University Guangzhou China

**Keywords:** Cervical lymph node, head and neck cancer, metastasis, radiotherapy, survival, unknown primary site

## Abstract

Despite advances in diagnosis and treatment, the existence of cervical lymph node carcinoma of unknown primary site (CCUP) has always been an urgent problem worldwide. There is still no consensus on the optimal management for CCUP. In this retrospective review, we analyze the clinical characteristics of CCUP patients treated at our institution and examine how these characteristics and treatments were associated with survival. Clinicopathologic features, treatments, and survival outcomes of 154 CCUP patients were collected from the hospital records and analyzed. Survival was estimated by Kaplan–Meier methods and compared by the log‐rank test. Cox proportional hazards regression analysis was used to assess the factors independently associated with overall survival (OS) and progression‐free survival (PFS). Median follow‐up period was 26.44 months (range, 0.53–146.53 months). Multivariate analysis showed N stage, pathologic type, and lymph node extranodal extension (ENE) to be independent prognostic factors for OS in CCUP patients, but not PFS. Subgroup analysis of patients who received radiotherapy showed that radiotherapy to the pharyngeal mucosa was associated with better OS (*P *=* *0.045), but not with better PFS. Advanced N stage, nonsquamous cell carcinoma, and lymph node ENE predict poor prognosis in patients with CCUP. In addition, radiotherapy to suspicious mucosa is accompanied by better OS. These study findings should be useful to clinicians when selecting the treatment approach.

## Introduction

Carcinoma of unknown primary site (CUP) is a form of cancer that the patient presents with lymph nodal or distant metastases but no obvious primary 1. The appropriate treatment strategy can be selected only after definitive diagnosis, but often the primary cannot be identified even after long follow‐up and the application of advanced diagnostic methods such as positron emission tomography. Interestingly, however, there has been an obvious decrease in the incidence of CUP over the last decade 2.

Cervical carcinoma of unknown primary site (CCUP) accounts for 2–5% of all head and neck cancers 3. Squamous cell carcinoma is the most common histologic type, accounting for 75% of cases, followed by adenocarcinoma, undifferentiated carcinoma, and other malignancies 4. Diagnostic workup of CCUP should include physical examination, with thorough evaluation of the head and neck mucosa; biopsy of suspicious lymph nodes; pan‐endoscopy with randomized biopsy and unilateral or bilateral tonsillectomy; diagnostic imaging with computed tomography (CT), magnetic resonance imaging (MRI), or 18F‐fluorodeoxyglucose positron emission tomography (FDG‐PET) scan; and interdisciplinary consultations 5. The management of patients with CCUP remains controversial and undefined because of the wide variations in age at presentation, pathologic type, involved nodal area, extent of lymph node involvement, and even the response to different therapies. In general, management of CCUP depends on the pattern of cervical lymphatic metastasis, and treatment options include surgery, radiotherapy, or chemoradiotherapy. The decision on whether radiotherapy to the neck should cover the putative mucosal site is also based on the assessment of risk factors. A consensus on the correct approach to diagnosis and management of CCUP is essential to guide the clinician, but so far, no prospective studies or randomized clinical trials have examined and compared the efficacies of different treatments.

The aim of this retrospective review was to analyze the clinical characteristics of CCUP and to determine how clinical characteristics and different treatments were associated with long‐term outcomes.

## Methods

### Patients

Between 2003 and 2014, 154 patients with CCUP were admitted and treated at the Sun Yat‐sen University Cancer Center (SYSUCC). Sun Yat‐sen University Cancer Center is the largest integrated center in South China for cancer‐related care, education, research, and prevention. In addition with a comprehensive range of healthcare services for cancer diagnosis and treatment (capacity over 1000 beds), NPC has a very high incidence in South China. And this disease is thus extensively researched in SYSUCC, where every year about 3000 new NPC patients are treated. NPC and CCUP are both with the neck mass as the first complaint, so there are relatively many CCUP patients diagnosed in our center.

The demographic and clinical data (including follow‐up data) of these patients were retrieved from the electronic medical records and retrospectively reviewed. The inclusion criteria were as follows: (1) appearing the neck mass as the initial and the only complaint; (2) undiscovering the primary site after comprehensive diagnostic workups, including pathologic immunohistochemical molecular biological and some other special methods; (3) no history of other tumors; no history of removal of suspicious tumor; (4) not appearing the primary site during the therapy; (5) newly pathologically diagnosed with cancer; and (6) complete follow‐up data. This study was approved by the Research Ethics Committee of Sun Yat‐sen University Cancer.

### Diagnostic workup and staging

Diagnostic workup included complete medical history, physical examination, and hematological examination including tumor biomarker, anti‐Epstein–Barr virus antibody, and Epstein–Barr virus DNA. Endoscopic examination includes direct nasopharyngoscopy, fiber‐optic laryngoscopy, esophagogastroscopy, and bronchoscopy. Imageological examination includes MRI of the head and neck and chest regions, contrast‐enhanced CT of chest, abdomen, and pelvic cavity, and upper gastroenterography. In addition, bone scan and 18F‐FDG‐PET were also performed more often. All patients conducted pathologic diagnosis of metastatic lymph node. Lymph node biopsy procedures included the following: fine needle aspiration (FNA), 86 patients; incisional biopsy, 6 patients; excisional biopsy, 28 patients; core needle biopsy, 9 patients; and neck dissection, 25 patients.

All patients presented with cervical nodes. N staging (N1, N2, or N3) was according to the American Joint Committee for Cancer (AJCC) staging system. Overall stage was defined as III (N1, N2) or IV (N3, M1).

### Treatment

The treatment modalities included surgery, radiotherapy, chemotherapy, or palliative treatment. For patients with squamous cancer staged as N1 without ENE, and without lymph node excision or biopsy, simple surgery or radiotherapy was considered; for those patients with well‐differentiated squamous cancer with ENE, with lymph node excision or biopsy, with surgical residues, or with N2–3 stage, surgery followed by radiotherapy was preferred. While, the patients with poorly differentiated or undifferentiated, we could conduct radiotherapy first, followed by surgery if tumor remain. When pathologically diagnosed with metastatic adenocarcinoma, surgery was considered as the main therapy, bilateral thyroidectomy was conducted when there existed with the possibility of thyroid source.

Surgery consisted of unilateral or bilateral neck dissection, radical neck dissection, or modified radical neck dissection, according to the location of lesion. Surgery was followed by radiotherapy and chemotherapy to treat residual nodes. The use of three‐dimensional conformal radiotherapy (3D‐CRT) and intensity‐modulated radiotherapy (IMRT) offers the target volume coverage of the entire neck. Radiotherapy was conducted alone to the unilateral neck, bilateral neck, and putative or suspicious mucosa (nasopharynx, oropharynx, and hypopharynx) plus bilateral neck. With standard fractionated radiotherapy of 2 Gy per fraction and 5 fractions per week, the total dose to the sites was 60–70 Gy. For postoperative cases, depending on the surgical margin status, 60–66 Gy was prescribed to the surgical nodal bed. The parotids, cervical esophagus, spinal cord, brain stem, optic nerves, and the orbits are also outlined as dose‐limiting structures. Concurrent chemotherapy was administered for patients with more advanced disease stages to improve local control and reduce the risk of distant spread. Neoadjuvant chemotherapy schedule included different combinations of taxanes, 5‐flurouracil, and platinum‐based drugs. Concomitant chemotherapy schedule consisted of cisplatin/carboplatin with or without 5‐fluorouracil. Cetuximab was added in two patients as adjuvant therapy.

### Follow‐up

Median follow‐up time was for 26.44 months (range, 0.53–146.5 months). Follow‐up evaluations were performed every 3 months in the first 3 years, every 6 months in the following 3–5 years, and annually thereafter until death. At the follow‐up visit, all patients received clinical, endoscopic, ultrasound, and radiological examinations (MRI, CT, bone scan).

The primary end point was overall survival (OS) and the secondary end point progression‐free survival (PFS). OS was calculated from the date of initial diagnosis to the date of death from any cause or patient censoring at the last follow‐up. PFS was determined from the date of initial diagnosis to the date of recurrence or distant relapse or patient censoring at the last follow‐up.

### Statistical analysis

Survival outcomes were analyzed using the Kaplan–Meier method, and comparisons were made using the log‐rank test. Variables that were significant in the univariate tests were entered into multivariate regression analysis performed using the Cox proportional hazards model and the stepwise method. Hazard ratios (HRs) with 95% confidence intervals (CI) were calculated for each independent factor. Statistical analysis was performed using IBM SPSS version 22.0 (IBM Corp., Armonk, NY). All significance tests were two‐sided; *P *<* *0.05 was defined as statistically significant.

## Results

### Baseline characteristics of the study population and subgroups

A total of 154 patients (74% males) with pathologically confirmed diagnosis of cancer were included in the study. The median age was 50 years (range, 14–75 years). Table 1 lists the demographic and clinical characteristics of the patients. The most common histopathology was squamous cell carcinoma 101 (65.6%), followed by adenocarcinoma 28 (18.2%) and others (adenocystic carcinoma, fusocellular sarcoma, mucoepidermoid carcinoma, neuroendocrine carcinoma, malignant melanoma, lymphoid/epithelioid carcinoma, and small cell carcinoma; 16.2%). N1 stage was seen in 78 (50.6%) patients, N2 stage in 62 (40.2%), and N3 stage in 14 (9.2%). Lymph node extranodal extension (ENE) was present in 56 (36.4%) patients and distant metastasis at diagnosis in 3 patients. Overall stage was III (N1) in 78 (50.6%) patients and IV (N2–3, M1) in 76 (49.4%) patients. With regard to treatment, 67 (43.5%) patients received neck dissection, 68 (44.2%) received chemotherapy, and 42 (27.3%) received radiotherapy (23 3D‐CRT and 19 IMRT).

**Table 1:** Baseline characteristics of the 154 patients with CCUP

Characteristics	*n* (%)
Total	154 (100)
Gender	
Male	114 (74.0)
Female	40 (26.0)
Age (year)	
<50	70 (45.5)
≥50	84 (54.5)
Smoking	
No	95 (61.7)
Yes	59 (38.3)
Drinking	
No	122 (79.2)
Yes	32 (20.8)
Overall stage	
III	78 (50.6)
IV	76 (49.4)
N category	
N1	78 (50.6)
N2	62 (40.2)
N3	14 (9.2)
Lymph node ENE	
No	98 (63.6)
Yes	56 (36.4)
Pathologic type	
Squamous cell carcinoma	101 (65.6)
Adenocarcinoma	28 (18.2)
Other types^a^	25 (16.2)
Therapy	
Surgery	67 (43.5)
Radiotherapy	42 (27.3)
Chemotherapy	68 (44.2)
Death	
No	103 (66.9)
Yes	51 (33.1)
Locoregional recurrence	
No	145 (94.2)
Yes	9 (5.8)

CCUP, cervical lymph node carcinoma metastases from unknown primary site; ENE, extranodal extension; N, node; NPC, nasopharyngeal carcinoma.

a Other types: adenocystic carcinoma, fusocellular sarcoma, mucoepidermoid carcinoma, neuroendocrine carcinoma, malignant melanoma, lymphoid/epithelioid carcinoma, small cell carcinoma.

During follow‐up, in all, 51 (33.1%) patients died, 13 patients experienced distant metastasis, and 9 (5.8%) patients developed regional recurrence.

### Factors associated with OS and PFS

**Figure 1:**
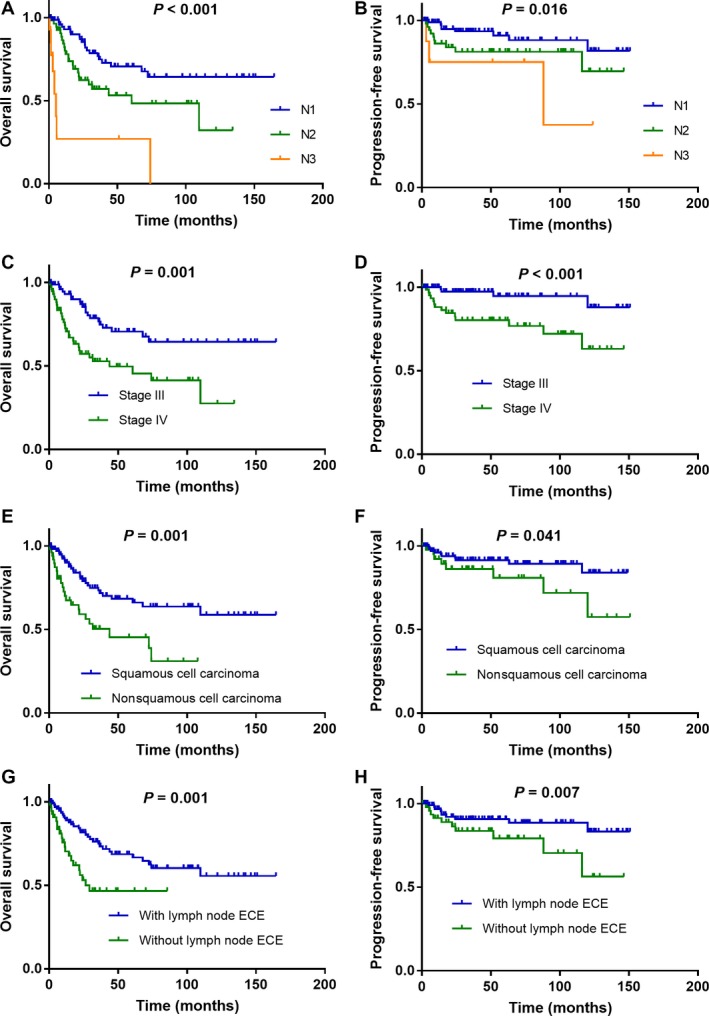
Kaplan–Meier overall survival and progression‐free survival curves for all 154 patients with CCUP stratified by N category (A, B), overall stage (C, D), pathologic type (E, F), and with or without lymph node ENE (G, H).

Median follow‐up duration was 26.44 months (range, 0.53–164.53 months). The 2‐year and 5‐year OS rates were 73.5% and 59.4%, respectively, and the PFS rates were 87.6% and 84.0%, respectively. In survival analysis, higher N stage was associated with poorer OS and PFS compared with the less advanced stage (log‐rank test *P *<* *0.001 and *P *=* *0.016, respectively; Fig. 1A and B). Similarly, overall stage was also associated with poorer OS and PFS (*P *=* *0.02 and *P *<* *0.001, respectively; Fig. 1C and D). Compared with squamous carcinoma, nonsquamous cell carcinoma presented poorer OS and PFS (*P *=* *0.001 and *P *=* *0.041, respectively; Fig. 1E and F). Lymph node ENE was also associated with poorer OS and PFS (*P *=* *0.001 and *P *=* *0.007, respectively; Fig. 1G and H).

### Independent prognostic factors for OS and PFS

Univariate analysis showed several clinicopathologic variables to be associated with OS. After excluding correlated variables, N category, overall stage, pathologic type, lymph node ENE, and treatment administered (radiotherapy, chemotherapy, or surgery) were tested in multivariate analysis. Independent predictors of OS were N stage (*P *<* *0.001; N2 vs. N1: HR 1.890, 95% CI 1.017–3.512; *P *=* *0.044; N3 vs. N1: HR 7.243, 95% CI 3.033–17.299; *P *<* *0.001); lymph node ENE (HR 2.157, 95% CI 1.168–3.985; *P *=* *0.014), and pathologic type (HR 1.977, 95% CI 1.102–3.546; *P *=* *0.022; Table 2), but they were not independent prognostic factors for PFS.

**Table 2:** Multivariate Cox proportional hazards regression model of OS for the 154 patients with CCUP

Variable	HR (95% CI)	*P*
N category		
N1	1 (reference)	<0.001
N2 vs. N1	1.890 (1.017–3.512)	0.044
N3 vs. N1	7.243 (3.033–17.299)	<0.001
Lymph node ENE		
Yes vs. no	2.157 (1.168–3.985)	0.014
Pathologic type		
Squamous cell carcinoma vs. other types	1.977 (1.102–3.546)	0.022

HR, hazard ratio; CI, confidence interval.

### Subgroup analysis of patients treated with radiotherapy

**Figure 2:**
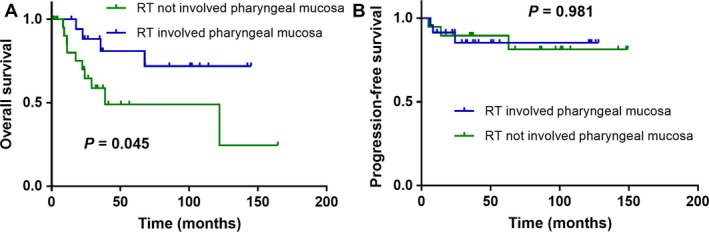
Kaplan–Meier overall survival (A) and progression‐free survival (B) curves for subgroup of 42 patients who received radiotherapy with CCUP stratified by RT involved pharyngeal mucosa or not.

Subgroup analysis was performed to assess the association between clinical factors and survival in 42 patients treated with radiotherapy. In this subgroup, the 5‐year OS and PFS rates were 64.2% and 87.3%, respectively. Nineteen patients received nasopharynx and/or oropharynx mucosa radiotherapy. In survival analysis, radiotherapy to pharyngeal mucosa was shown to be associated with better OS (*P *=* *0.045; Fig. 2A), but not with better PFS (*P *=* *0.981; Fig. 2B).

## Discussion

The present study was performed to identify the clinical characteristics of CCUP patients in our institution and to assess the association of these factors with survival. In this limited sample, patients with early stage disease, those without lymph node ENE and squamous carcinoma histopathology, and those undergoing therapy of radiotherapy, chemotherapy, and surgery have better OS and PFS. Here, the emphasis is on the value of radiotherapy in the treatment of CCUP which is the most groundbreaking aspect in the era of emerging therapy.

So far, there is no worldwide consensus on the treatment of CCUP. In the numerous retrospective studies that have been published, lymph node dissection followed by adjuvant radiotherapy or chemoradiotherapy, according to the N stage, is the most commonly adopted approach. However, anti‐neoplastic treatment is associated with many adverse effects. Advanced radiotherapy techniques such as IMRT are superior to conventional radiotherapy in terms of target coverage and sparing of organs at risk. Previous research has shown that IMRT for CCUP results in lower early and late toxicities (dysphagia, xerostomia, dysphagia, or skin fibrosis) than conventional radiotherapy, while providing equivalent efficacy 6, which was partly consistent with our results. Of 42 patients treated with radiotherapy in our study, 23 received 3D‐CRT and 19 received IMRT. The dose, usually given with standard fractionation (dose per fraction of 1.8–2.0 Gy), was 65–70 Gy for the involved nodal stations and 50 Gy for the uninvolved neck and mucosal sites, according to the level of the neck affected. In the case of clinically suspicious mucosal sites, a dose of 60–64 Gy was administered. The survival outcomes were not significantly different between patients treated by the two techniques. The 5‐year OS rate of CCUP patients was much lower than that of primary head and neck carcinoma patients (60% vs. 80–90%) 7; the difference may owe to the lower radiosensitivity of CCUP and indirectly indicates the difference in biological characteristics.

For the aspects of the radiotherapeutic extent, optimal extension of radiotherapy volumes which is the most important remains an issue and the bias always exists. A study comparing survival in CCUP patients receiving ipsilateral radiotherapy and comprehensive radiotherapy (i.e., including the potential mucosal surfaces and ipsilateral or bilateral neck) reported that there was no difference in OS and disease‐free survival between the two methods 8. A retrospective comparison of bilateral neck radiotherapy with unilateral neck radiotherapy also did not show significant differences in terms of locoregional control and survival; the 5‐year OS rates were 22% after unilateral irradiation vs 23% after bilateral radiotherapy (*P *=* *0.944) 9. IMRT delivered to comprehensive bilateral neck and putative mucosal site (including nasopharynx, oropharynx, and retropharyngeal lymph nodes) appears to be effective for patients with head and neck CUP, but did not compromise locoregional control 10. Some reviews have suggested that nodal resection and bilateral neck radiotherapy provide better regional control than ipsilateral neck radiotherapy 11, 12. It was revealed that treatment with curative intent and extensive irradiation of bilateral neck and pharyngeal mucosa was favorable prognostic factors for the patients with CCUP, which resulted in significantly better outcomes 13. Many authors have observed that mucosal irradiation results in lower likelihood of the primary tumor being identified and also reduces the risk of regional recurrence, but it does not improve the OS rate. Extended‐volume radiotherapy (i.e., to putative mucosal sites and bilateral neck) results in lower locoregional failure than volume‐restricted radiotherapy (ipsilateral neck) (27% vs. 51%), but the rates of overall survival and disease‐free survival and the emergence of the primary cancer are similar for both radiotherapy approaches 14, and therefore, no definite conclusions can be drawn on this issue. As mentioned before about our study, radiotherapy to pharyngeal mucosa was associated with improved OS and PFS. With modern techniques, 5‐year OS and PFS were 59.4% and 84.0%, respectively, much higher than that achieved with older diagnostic and therapeutic methods.

There have only been a few studies examining the possible mechanisms for the occurrence of CCUP. Why the metastatic site appears before the primary site does is not known, and there is still no method for locating the primary tumor. Information on the primary site is unlearned and has scarcely been analyzed. A review of several phase II or III clinical trials was conducted on CUP, and using molecular/genetic traits could distinct from tumors of known primaries through a distinct biological behavior, although tumor shrinkage and median survival in CUP patients were similar to those of patients with metastatic cancer 15.

A study combined p16 and p53 expressions in CCUP revealed that patients with both p16‐negative and p53‐positive tumors showed a significantly poorer tumor‐specific survival (TSS) compared to those with either p16+/p53−, p16+/p53+, or p16−/p53−, which may represent as a method for risk stratification 16. Over the last decade, the prevalence of HPV‐associated squamous cell cancer has increased, and immunostaining for the p16 protein has become a reliable surrogate marker for HPV infection 17. p16‐positive lymph node metastasis strongly suggests an occult primary lesion in the oropharynx 18. A systematic review 19 further stated that HPV status could be routinely assessed in CCUP patients as it may lead to revealing the primary tumor and may even alter the decision to select treatment, while there was a much lower incidence of HPV in China compared to Western countries. The patients in our study rarely received HPV or p16 detection; thus, we have not analyzed the correlations between HPV and clinical outcomes of CCUP.

Even some reports attempted to screen for similarities and differences in incidence patterns between tumors of profiling of multiple gene expression via autopsy or molecular array profiling 20. Keeping up with the pace of emerging therapeutic era, there was a hypothesis that primary lesions may be influenced by a variety of immune‐active cell‐mediated immune function, therefore, in a relatively static or slow growth state, and metastases clone and grow rapidly in the appropriate environment. In addition, the intricate anatomy of the head and neck region also helps keep the primary tumor hidden.

Therapeutic or prognostic benefits depend on the early identification of the primary and that will be possible only after the molecular mechanisms responsible for primary tumor dormancy and early metastatic spread are clarified. It was an opportunity also a challenge when although the most active (taxane, platinum, anthracycline) or targeted (bevacizumab, erlotinib) drugs were combined, disease control was not improved as identified in the previous systematic review 15. Thus, considering the limitations of therapy, plus the tumor specificity, the deeper exploration for all the biased aspects of CCUP should be on agenda as soon as possible.

## Study Limitations

This study has some limitations. First, the study included patients treated between 2003 and 2014, over which period there have been many changes in diagnostic and therapeutic approaches; this may have influenced the survival analysis. Also, some patients have lost follow‐up and died from unknown causes. Second, HPV associated with head and neck cancer has been certified lately, and the lack of HPV detection in the study is definitely a flaw in our study. Third, only 42 of the 154 patients received radiotherapy; subgroup analysis showing that radiotherapy to the pharyngeal mucosa was associated with better OS may not be reliable because of the small sample size. Moreover, the effects of radiotherapy may have been differently influenced by the chemotherapeutic combination used. Despite these limitations, this study highlights the need to prospectively validate the impact of prognostic factors and new treatments on patient outcome in CCUP and to explore the mechanisms underlying the occurrence of CCUP.

## Conclusions

This study showed that advanced N stage, nonsquamous cell carcinoma histopathology, and lymph node ENE are predictive of poor prognosis in CCUP and also that radiotherapy to suspicious mucosa is associated with better OS. These findings can help guide treatment in patients when the primary is not identified despite thorough examination and investigations. Large prospective studies and randomized trials are needed to confirm these findings and clarify underlying mechanisms.

## Conflict of Interest

The authors declare that they have no competing interests.

## Data Accessibility

The authenticity of this article has been validated by uploading the key raw data onto the Research Data Deposit public platform (http://www.researchdata.org.cn), and the RDD number is RDDA2018000509.
